# Evaluation of the Bio-Evolution Microsporidia generic and typing real-time PCR assays for the diagnosis of intestinal microsporidiosis[Fn FN1]

**DOI:** 10.1051/parasite/2022055

**Published:** 2022-11-25

**Authors:** Maxime Moniot, Céline Nourrisson, Virginie Bonnin, Céline Damiani, Nicolas Argy, Julie Bonhomme, Emilie Fréalle, Cécile Angebault, Anne Debourgogne, Emilie Sitterlé, Pierre Flori, Julie Brunet, Frédéric Dalle, Loïc Favennec, Philippe Poirier

**Affiliations:** 1 Laboratoire de Parasitologie-Mycologie, CHU Clermont-Ferrand, 3IHP 63003 Clermont-Ferrand France; 2 Microbes, Intestin, Inflammation et Susceptibilité de l’Hôte (M2iSH), UMR Inserm/Université Clermont Auvergne U1071, USC INRA 2018 63000 Clermont-Ferrand France; 3 Laboratoire de Parasitologie et Mycologie Médicales, CBH, CHU Amiens Picardie; Equipe Agents Infectieux, Résistance et Chimiothérapie (AGIR) UR4294, Université de Picardie Jules Verne 80480 Amiens France; 4 Service de Parasitologie Mycologie, CHU Bichat-Claude-Bernard, Assistance Publique des Hôpitaux de Paris (APHP); IRD UMR MERIT 261, Faculté de Pharmacie, Université de Paris Cité 75018 Paris France; 5 Service de Microbiologie, CHU Caen, ToxEMAC-ABTE, Normandie Univ, Unicaen & Unirouen 14033 Caen France; 6 Laboratoire de Parasitologie et Mycologie Médicale, CHU Lille 59037 Lille France; 7 Unité de Parasitologie-Mycologie, Département de Prévention, Diagnostic et Traitement des Infections, CHU Henri Mondor, AP-HP; EA DYNAMiC 7380, Faculté de Santé, Univ Paris-Est Créteil 94000 Créteil France; 8 Laboratoire de Microbiologie, CHU Nancy 54035 Nancy France; 9 Unité de Parasitologie-Mycologie, Service de Microbiologie clinique, GHU Necker-Enfants-Malades, Assistance Publique des Hôpitaux de Paris (APHP) 75743 Paris France; 10 Laboratoire de Parasitologie Mycologie, CHU Saint-Etienne 42055 Saint-Etienne France; 11 Laboratoire de Parasitologie et de Mycologie Médicale, Plateau Technique de Microbiologie, Hôpitaux Universitaires de Strasbourg 67091 Strasbourg France; 12 Laboratoire de Parasitologie-Mycologie, Plateforme de Biologie Hospitalo-universitaire CHU Dijon; UMR PAM Univ Bourgogne Franche-Comté – AgroSup Dijon – Equipe Vin, Aliment, Microbiologie, Stress 21079 Dijon France; 13 Service de Parasitologie Mycologie, CHU Rouen; EA ESCAPE 7510, Université de Rouen Normandie 76031 Rouen France; 14 CNR LE Cryptosporidioses, Santé Publique France 76031 Rouen France

**Keywords:** Microsporidiosis, Real-time PCR, Bio-Evolution, *Enterocytozoon bieneusi*, *Encephalitozoon intestinalis*

## Abstract

Cases of intestinal microsporidiosis infection are underestimated and affect both immunocompromized and immunocompetent patients. Real-time PCR is superseding microscopic examination for its diagnosis in medical analysis laboratories. However, few manufacturers include microsporidia in their PCR panel for the diagnosis of infectious gastroenteritis. Here, we evaluated the performances of the real-time PCR assays microsporidia generic and microsporidia typing (Bio-Evolution, France) on the Rotor-Gene Q real-time PCR cycler (Qiagen, France). We included 45 negative and 44 positive stool samples for *Enterocytozoon bieneusi* (*n* = 34, with various genotypes), *Encephalitozoon intestinalis* (*n* = 4), *Encephalitozoon hellem* (*n* = 4), and *Encephalitozoon cuniculi* (*n* = 2). We also studied a four-year survey of an inter-laboratory quality control program including 9 centers that used this commercial assay. Sensitivity and specificity of the microsporidia generic assay were 86.4% and 93.3%, respectively. *Encephalitozoon hellem* and *Encephalitozoon cuniculi* were detected by the microsporidia generic PCR assay but not by the microsporidia typing PCR assay. These results were consistent with the results of the inter-laboratory quality control program. In conclusion, Bio-Evolution Real-time PCR assays are useful tools for intestinal microsporidiosis, but negative results for microsporidia typing assays require supplementary analyses to confirm *E. hellem* or *E. cuniculi* infections.

## Abstract (fr)

Les microsporidioses intestinales sont des infections sous-estimées affectant à la fois les patients immunodéprimés et immunocompétents. Le diagnostic microscopique en laboratoire médical est aujourd’hui supplanté par la PCR en temps réel. Cependant, peu de fabricants incluent les microsporidies dans leurs panels PCR pour le diagnostic des gastro-entérites infectieuses. Ici, nous avons évalué les performances des tests PCR en temps réel microsporidia generic et microsporidia typing (Bio-Evolution, France) sur le thermocycleur PCR en temps réel Rotor-Gene Q (Qiagen, France). Nous avons inclus 45 échantillons de selles négatifs et 44 échantillons positifs pour *Enterocytozoon bieneusi* (*n* = 34, avec divers génotypes), *Encephalitozoon intestinalis* (*n* = 4), *Encephalitozoon hellem* (*n* = 4) et *Encephalitozoon cuniculi* (*n* = 2). Nous avons également analysé les résultats sur 4 ans d’un programme de contrôle qualité inter-laboratoires dont 9 centres ont utilisé ces kits commerciaux. La sensibilité et la spécificité du kit microsporidia generic étaient respectivement de 86,4 % et 93,3 %. *Encephalitozoon hellem* et *E. cuniculi* ont été détectés par le kit microsporidia generic mais pas par le kit microsporidia typing. Ces résultats étaient cohérents avec ceux du programme de contrôle de qualité inter-laboratoires. En conclusion, les tests de PCR en temps réel Bio-Evolution sont des outils intéressants pour la microsporidiose intestinale, mais un résultat négatif pour le test de typage microsporidia nécessite une analyse supplémentaire pour confirmer les infections à *E. hellem* ou *E. cuniculi*.

## Introduction

Intestinal microsporidiosis cases are associated with severe and chronic diarrhea in immunocompromized patients [13]. They were first reported as opportunistic infections in HIV-infected patients in the 1980s and 1990s, but nowadays they are mostly diagnosed in other immunocompromized populations, such as patients with solid organ transplantation [3, 5, 6, 8, 11, 15]. *Enterocytozoon bieneusi* accounts for more than 90% of intestinal microsporidiosis cases; the remaining cases involve *Encephalitozoon intestinalis, Encephalitozoon hellem* and *Encephalitozoon cuniculi* [13]. For a long time, the diagnosis of intestinal microsporidiosis was exclusively based on microscopy. In recent years, microscopy was superseded by real-time PCR for these diagnoses. Molecular tools allow for more sensitive diagnosis and give rapid species identification which is essential for therapeutic care, since treatment depends on the species [9, 13, 27]. Several in-house PCR methods have been published, targeting *E. bieneusi* and/or *E. intestinalis* [7, 17–19, 23, 29, 30, 32]. These targets are also included in ready-to-use commercial real-time PCR panels, some of which also enable detection of other gastrointestinal pathogens: ParaGENIE Cryptosporidia Microsporidia real-time PCR kit (Ademtech, France), Allplex GI-Helminth real-time PCR assay (Seegene, Germany), Novodiag^®^ Stool Parasites (Mobidiag, Finland), and the combination of two real-time PCRs: microsporidia generic assay (MGa) plus microsporidia typing assay (MTa) (Bio-Evolution, France). Only the Seegene and Ademtech panels have been evaluated to date, showing good and comparable performances to in-house real-time PCR assays for microsporidiosis diagnosis [2, 16, 20]. Regarding the Bio-Evolution assay, it has the particularity of proceeding in 2 steps. An initial assay (MGa) allows for the screening of stool samples and detects simultaneously *E. bieneusi* and *Encephalitozoon* species, but does not discriminate between them. The positive samples are then tested with the second assay (MTa) in order to specify whether they are *E. bieneusi* or *E. intestinalis*.

The main objective of this study was to assess performances of both Bio-Evolution Microsporidia generic and typing PCR assays with (i) microsporidia-negative and -positive stools, and (ii) a national interlaboratory quality control program.

## Material and methods

### Clinical samples

A total of 89 DNA extracts from fecal specimen were used in this study. For *E. cuniculi*, spores purified from an *in vitro* culture were spiked into a negative stool. Among them, 44 positive samples for microsporidia were obtained from the French Microsporidiosis Network, *i.e.*, initial diagnosis was made in each collaborative center by microscopy and/or real-time PCR and further confirmed by in-house real-time PCR according to Moniot *et al.*, with median cycle threshold (*Ct*) values of 27 (range, 18–38) (Supplementary Table 1) [19]. The different species and genotypes included are listed in Table 1. No co-infection with other pathogens was reported. For *E. bieneusi-*positive samples, genotype was identified by sequencing the Internal Transcribed Spacer (ITS) region, as previously described [14, 28]. *Encephalitozoon* species were also characterized by ITS sequencing. In all, 45 samples negative for microsporidia (checked by in-house real-time PCR according to Moniot *et al.*, [19]) were collected in the Clermont-Ferrand University Hospital, among which microscopic examination was negative for 20 samples and positive with other eukaryotic microorganisms for 25 samples (for more details see Table 1).

**Table 1:** Clinical stool samples used in this study.

Positive samples			Negative samples	
Species	Genotype	Number of samples	Species	Number of samples
*Enterocytozoon bieneusi* (*n* = 34)	A	4	Negatives	20
	C	8	*Cryptosporidium parvum*	2
	CAF-1	1	*Cryptosporidium hominis*	2
	C-like01	2	*Blastocystis* sp.	5
	C-like02	2	*Endolimax nana*	2
	C-like03	1	*Entamoeba coli*	2
	D	4	*Entamoeba histolytica*	2
	HND-I	1	*Entamoeba dispar*	2
	IV	3	*Giardia intestinalis*	2
	Wildboar2	1	*Candida albicans*	2
	Wildboar3	6	*Geotrichum candidum*	2
	WR5-like01	1	*Saccharomyces cerevisiae*	2
*Encephalitozoon intestinalis*		4	Total	45
*Encephalitozoon hellem*		4		
*Encephalitozoon cuniculi*		2		
Total		44		

### DNA extraction

DNA extractions were performed within the next few days following sample collection (samples were stored at 4 °C until DNA extraction). Two hundred mg of stool were placed in vials containing 800 μL of easyMAG lysis buffer (bioMérieux, France) and 100 μL of 0.5 mm glass beads (Next Advance, France). Then, a bead beating step was performed for 3 min at 3000 Hz (TissueLyser, Qiagen, France), followed by centrifugation for 10 min at 20,000 ×*g*. Then, DNA from 200 μL of the supernatant was extracted using a QIAamp Fast DNA Stool Mini Kit (Qiagen, France). DNA extracts were aliquoted to prevent several cycles of freezing/thawing and stored at −80 °C during the study (*i.e.*, three years maximum).

### Real-time PCR

The Bio-Evolution Microsporidia generic assay (MGa) and microsporidia typing assay (MTa) were performed on the Rotor-Gene Q thermocycler (Qiagen), according to the manufacturer’s instructions. For both MGa and MTa, the primers and probes target the 18S rRNA encoding gene. For MGa, the ampliﬁcation consisted in an initial denaturation step of 15 min at 95 °C, followed by 40 cycles of denaturation at 95 °C for 15 s, and annealing/elongation at 62 °C for 1 min. For MTa, the ampliﬁcation consisted in an initial denaturation step of 15 min at 95 °C, followed by 45 cycles of denaturation at 95 °C for 15 s, and annealing/elongation at 60 °C for 1 min. MGa includes an internal control for assessing the presence of PCR inhibitors, MTa does not. Sensitivity, specificity, and predictive positive and negative values claimed by the manufacturer are 88%, 83%, 96% and 63% for MGa, and 86% (*E. bieneusi*)/100% (*E. intestinalis*), 100% (both *E. bieneusi* and *E. intestinalis*), 100% (both *E. bieneusi* and *E. intestinalis*) and 63% for MTa, respectively. PCR results were given as positive or negative. As recommended by the manufacturer, results were considered positive when the *Ct* value was less than or equal to 38 for MGa and 40 (or between 40 and 45 twice consecutively) for MTa. For MGa-positive samples, *Ct* values were also recorded. Positive samples for MGa were submitted to MTa, according to the manufacturer’s recommendations. A positive MTa identifies both *E. bieneusi* and *E. intestinalis* in a sample. A negative MTa should be interpreted as presence of microsporidia DNA other than *E. bieneusi* or *E. intestinalis*.

### Interlaboratory quality control program

Each year, DNA samples from positive or negative microsporidia stool samples obtained by the same protocol mentioned before were sent at −20 °C to several medical analysis laboratories in France participating in a national external quality assessment, proposed by the Parasitology-Mycology unit of the Clermont-Ferrand University Hospital (France, coordinating center of the French Microsporidiosis Network). Four years of this program (2018–2021) were analyzed with laboratories using the Bio-Evolution PCR assays; this corresponded to nine samples and nine institutions.

### Results analysis and statistical considerations

Statistical analyses were performed with Excel software 2010. For PCR assay performances, sensitivity, specificity, and positive and negative predictive values were calculated considering qualitative results of MGa and MTa. For positive samples, MGa quantitative results (*Ct* values) were analyzed. Each discordant result was discussed regarding the *Ct* values and the species/genotype implicated.

## Results

### PCR assays performances

**Figure 1:**
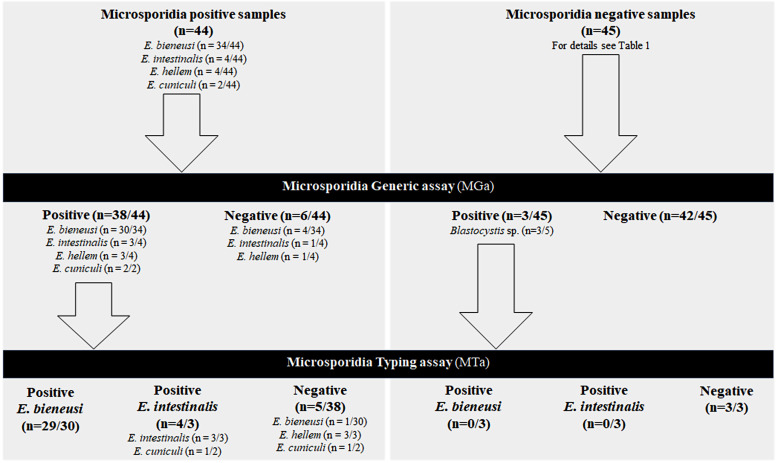
Study results flow chart. All samples (*n* = 89) were analyzed according to the manufacturer’s instructions, *i.e.*, each positive sample with the Microsporidia generic PCR assay (MGa) was submitted to the Microsporidia typing PCR assay (MTa).

The results of the study are presented in Figure 1. Among the 89 DNA tested, the MGa PCR assay was positive for 41 and negative for 48 samples. Three samples with *Blastocystis* sp. had a positive result with MGa. However, they were considered false positives because both in-house real-time PCR and end-point multiplex PCR (targeting *E. bieneusi* and *Encephalitozoon* spp. ITS), and microscopy examination, performed on these samples were negative. We did not observe any cross-reactivity with the other tested digestive pathogens or fungi commonly found in human stools (Table 1). Six samples had a false-negative result: four *E. bieneusi* (genotypes IV, *n* = 2; C, *n* = 1; Wildboar3, *n* = 1), one *E. intestinalis*, and one *E. hellem*. Overall sensitivity and specificity of MGa were 86.4% and 93.3%, respectively. Positive and negative predictive values were 92.7% and 87.5%, respectively (Table 2). The *E. intestinalis*, *E. hellem* and the four *E. bieneusi* isolates not detected with MGa had *Ct* values > 33 with our in-house PCR (Supplementary Table 1). In contrast, two *E. bieneusi* isolates with *Ct* values > 33 with our in-house PCR were detected by MGa (Supplementary Table 1). There was no association between *E. bieneusi* MGa false-negative and *E. bieneusi* genotype (A, IV, C and Wildboar3). Moreover, other positive samples with these genotypes were successfully detected in our study (Supplementary Table 1).

**Table 2:** Performances of the Microsporidia generic assay (MGa).

	Sensitivity % (CI95)	Specificity % (CI95)	Positive predictive value % (CI95)	Negative predictive value % (CI95)
Value (range)	86.4 (79.2–93.5)	93.3 (88.2–98.5)	92.7 (87.3–98.1)	87.5 (80.6–94.4)

Of the 41 MGa-positive samples, 33 were successfully identified with MTa including three *E. intestinalis* and 29 *E. bieneusi* belonging to various genotypes (A, C, CAF-1, C-like01, C-like02, C-like03, D, HND-I, IV, Wildboar2, Wildboar3, WR5-like01). As expected, MTa did not detect one isolate of *E. cuniculi* and three isolates of *E. hellem*. However, one *E. cuniculi* isolate gave a misidentification with a positive result on the *E. intestinalis* target. The three *Blastocystis* spp. that were positive with MGa were negative with MTa. The remaining five MTa-negative samples (among microsporidia positive samples) contained *E. bieneusi* (genotype A, *n* = 1), *E. cuniculi* (*n* = 1), and *E. hellem* (*n* = 3).

### Interlaboratory quality control program

**Figure 2:**
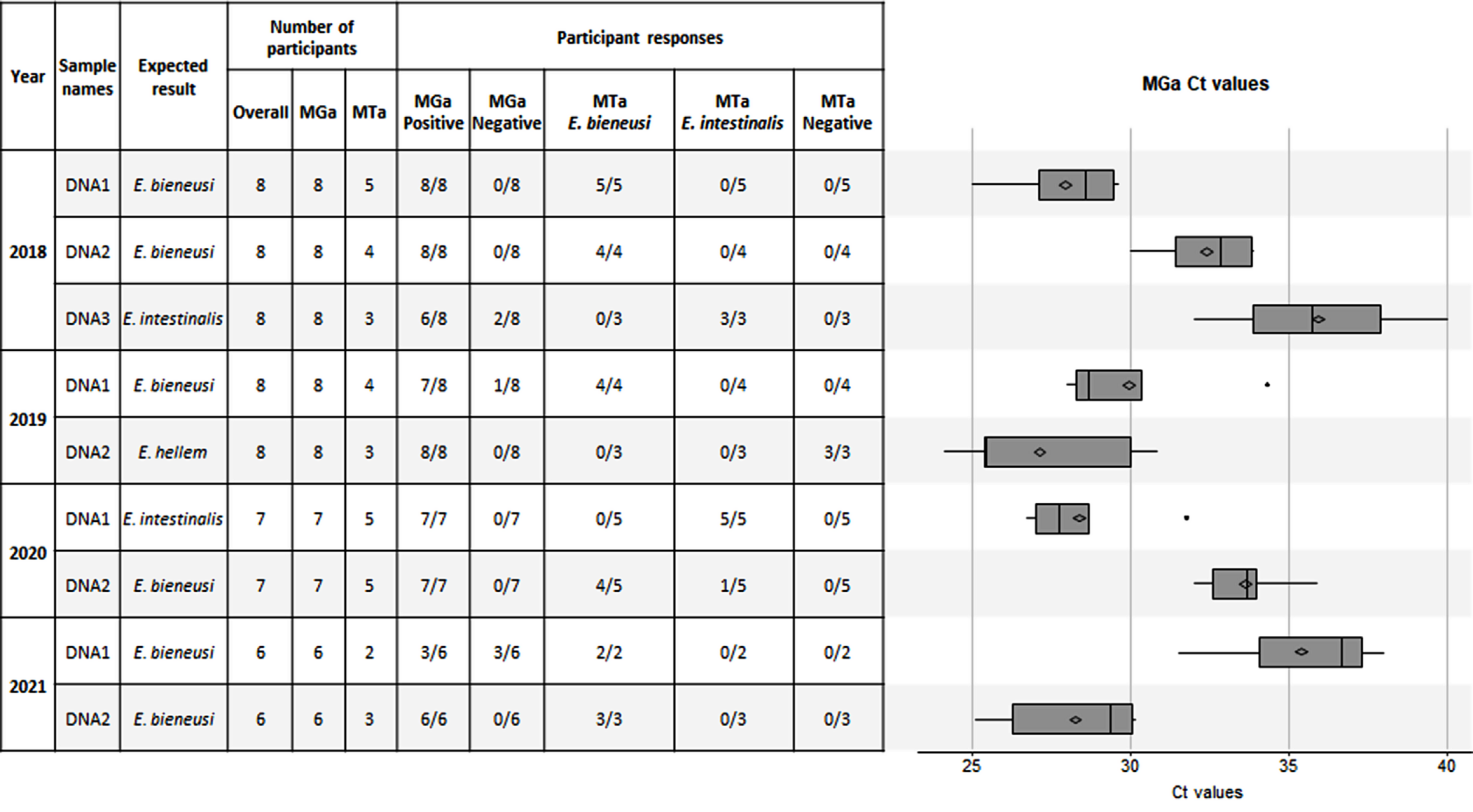
National interlaboratory quality control program: quantitative and qualitative results. Qualitative (left part) and quantitative (*Ct* values, right part) results of the Microsporidia generic PCR assay (MGa) and the Microsporidia typing assay (MTa) during the four-year survey.

Over the four-year multicenter survey (2018–2021), nine microsporidia-positive DNA samples were sent to participating laboratories. Participants using Bio Evolution assays performed PCR with various thermocyclers, including Rotor-Gene Q (Qiagen), Smartcycler (Cepheid), Lightcycler 480 (Roche diagnostics), ABI7500 or QuantStudio 5 or StepOnePlus Real-Time PCR System (ThermoFisher) and CFX96 (Bio-Rad). Qualitative and quantitative results are presented in Figure 2. Concerning qualitative results, four DNA samples (one *E. intestinalis* “2020-DNA1” and three *E. bieneusi* “2018-DNA1”, “2018-DNA2” and “2021-DNA2”) were correctly detected and identified by all laboratories. As expected, all laboratories detected the *E. hellem*-positive sample (“2019-DNA2”) with MGa, but were not able to determine the species with MTa. Four laboratories did not detect two *E. bieneusi*-positive samples (“2019-DNA1” and “2021-DNA1”) with MGa and two others an *E. intestinalis*-positive sample (“2018-DNA3”). Regarding the quantitative results, *Ct* values are scattered among laboratories. Of note, the “2018-DNA3” *E. intestinalis*-positive and the “2021-DNA1” *E. bieneusi*-positive samples had a low target amount.

## Discussion

Diagnosis of intestinal microsporidiosis in immunocompromized patient is increasing, especially in case of solid organ transplantation [3, 4, 8, 11, 15, 19]. Nowadays, real-time PCR assay is the best method to detect microsporidiosis in stool samples [9, 13, 27]. Numerous in-house PCR methods have been described and some commercial kits are available [1, 2, 7, 10, 16–20, 23, 29, 30, 32]. However, some of these have not been evaluated with clinical samples until now. In the Morio *et al.* (2019) study, the ParaGENIE Cryptosporidia Microsporidia real-time PCR kit showed sensitivity and specificity of 97.3% and 98.7% for *E. bieneusi* diagnosis, respectively [20]. Hence, the main objective of this study was to evaluate the performances of the two PCR assays commercialized by Bio-Evolution (MGa and MTa) with a panel of 44 microsporidia-positive DNA from clinical stool samples collected through the French Microsporidiosis Network. Of note, this study evaluates only the amplification step and not the extraction step which is known to be fundamental [33]. More generally, performances of molecular diagnostic techniques for intestinal parasites are highly dependent on stool sample pretreatment and the method used for DNA extraction [22]. Nevertheless, the manufacturer’s instructions did not include recommendations for these steps. One limitation when interpreting MGa and MTa performances for *Encephalitozoon* species was the difficulty in collecting enough positive samples for that genus. Because microsporidia are closely related to fungi, we recognize that it would have been relevant to test more fungal species during the cross-reactivity experiments. This will have to be addressed more deeply in future studies on molecular tools for the diagnosis of intestinal microsporidiosis. Overall sensitivity and specificity of MGa were 86.4% and 93.3%, respectively (Table 2 and Fig. 1). The absence of detection of some positive samples by MGa can be explained by a low load of microsporidia-DNA, as shown by the national interlaboratory quality control program. The “2018-DNA3” *E. intestinalis*-positive and the “2021-DNA1” *E. bieneusi*-positive samples had a low target amount, which could explain a poorer detection rate among participating laboratories (Fig. 2). Concerning the DNA load, *Ct* values varied greatly between laboratories for some samples, which can not only be explained by inter-operator variability. We hypothesized that the variability could be due to the thermocycler used, but this was not evaluated in our work because some centers did not specify the thermocycler used. For *E. bieneusi*-positive samples, it can also be hypothesized that sensitivity could depend on ITS genotypes. Of note, MGa targets the small subunit ribosomal RNA just before the ITS region. However, *E. bieneusi* discordant samples showed different genotypes (A, IV, C and Wildboar3). Moreover, other positive samples with these genotypes were successfully detected in our study (Supplementary Table 1). The lack of sensitivity seems to be correlated with low microsporidia-DNA quantity rather than a particular genotype of *E. bieneusi*, but the number of discordant samples is not sufficient to confirm this hypothesis. Interestingly, in some cases we observed discordances between MGa and MTa results. MGa detected *E. cuniculi* and *E. hellem* samples, but also some *Blastocystis* sp. samples. On the one hand, the PCR assay handbook clearly mentions that *Blastocystis* spp. may result in a flat curve with MGa, but are associated with an MTa-negative result, allowing microsporidiosis exclusion. On the other hand, *E. cuniculi* and *E. hellem* were not detected by MTa, which does not target these two species. These findings were confirmed by the qualitative results of the national interlaboratory quality control program. However, *E. cuniculi* and *E. hellem* are also responsible for intestinal microsporidiosis, even more frequently than *E. intestinalis* in some parts of the world [12, 21, 24–26, 31]. So, a positive MGa with an exponential curve associated with a negative MTa requires another confirmation assay to rule out *E. cuniculi* or *E. hellem* infections*.* This issue may also exist with other commercial kits: the ParaGENIE Cryptosporidia Microsporidia real-time PCR kit is able to differentiate between *E. bieneusi* and *E. intestinalis*, but there are no data about other *Encephalitozoon* species, and the Allplex GI-Helminth real-time PCR assay detects *E. bieneusi* and *Encephalitozoon* species without differentiation, which is inadequate for species-specific treatment (*i.e.*, fumagillin for *E. bieneusi* and albendazole for *Encephalitozoon* spp. infections) [2, 13, 16, 20]. Detection of and differentiation between *E. bieneusi* and *Encephalitozoon* spp. are possible with Novodiag^®^ Stool Parasites, although no evaluation has yet been published.

In conclusion, the Bio-Evolution Microsporidia generic and typing assays show good performances for *E. bieneusi* or *E. intestinalis* intestinal microsporidiosis diagnosis, as shown by our study in Clermont-Ferrand and the inter-laboratory quality control program. The two-step process is time consuming, but enables us to differentiate between *E. bieneusi* and *E. intestinalis* for specific treatment. Nevertheless, in case of discordant MGa/MTa results, additional analyses should be considered to detect/exclude other *Encephalitozoon* species.

## Supplementary material

The supplementary material of this article is available at https://www.parasite-journal.org/10.1051/parasite/2022055/olm.
Supplementary Table 1:
Quantitative results of our in-house PCR assay [19].
