# Supplementary material for: Evaluation of the Bio-Evolution Microsporidia generic and typing real-time PCR assays for the diagnosis of intestinal microsporidiosis
Source: Parasite. 2022 Nov 25;29:55. doi: 10.1051/parasite/2022055 (PMC9879149; doi:10.1051/parasite/2022055)
Supplement: Supplementary Table 1: — Quantitative results of our in-house PCR assay [19]. [file parasite-29-55-s1.pdf]

Supplementary Table 1. Quantitative results of our in-house PCR assay [19].

Number	Species	Genotype	In-house PCR assay result (Ct)	Microsporidia generic assay result
1	*E. bieneusi*	C-like02-NG	18	Positive
2		HND-I	20	Positive
3		C-like02-NG	21	Positive
4		Wildboar3	21	Positive
5		A	21	Positive
6		Wildboar3	22	Positive
7		C	22	Positive
8		D	22	Positive
9		Wildboar3	23	Positive
10		C-like03-NG	24	Positive
11		A	24	Positive
12		Wildboar3	24	Positive
13		C-like01-NG	25	Positive
14		Wildboar2	25	Positive
15		CAF-1	25	Positive
16		Wildboar3	26	Positive
17		IV	27	Positive
18		D	27	Positive
19		C	27	Positive
20		C	28	Positive
21		C	28	Positive
22		A	29	Positive
23		D	29	Positive
24		C	29	Positive
25		WR5-like-NG	30	Positive
26		C	30	Positive
27		D	30	Positive
28		C	31	Positive
29		IV	33	Negative
30		Wildboar3	34	Negative
31		A	34	Positive
32		IV	35	Negative
33		C	35	Negative
34		C-like01-NG	36	Positive
35	*E. cuniculi*	n.d.	19	Positive
36			19	Positive
37	*E. hellem*	n.d.	25	Positive
38			25	Positive
39			26	Positive
40			38	Negative
41	*E. intestinalis*	n.d.	25	Positive
42			31	Positive
43			31	Positive
44			35	Negative

n.d. : not determined
